# Multiscale Simulation of the Impact of Defects on Elevated-Metal Metal-Oxide IGZO TFTs

**DOI:** 10.3390/mi16020141

**Published:** 2025-01-25

**Authors:** Chuanxue Sun, Xiaoyu Dou, Zhichao Du, Haitao Dong, Xiaopeng Li, Pengpeng Sang, Xuepeng Zhan, Fei Mo, Jixuan Wu, Jiezhi Chen

**Affiliations:** 1 School of Information Science and Engineering (ISE), Shandong University, Qingdao 266237, China; 202232710@mail.sdu.edu.cn (C.S.);; 2 School of Integrated Circuits and Electronics, Beijing Institute of Technology, Beijing 100081, China

**Keywords:** elevated-metal metal-oxide (EMMO), IGZO, positive bias stress (PBS), oxygen vacancies

## Abstract

This study explores the impact of oxygen vacancy defects on elevated-metal metal-oxide (EMMO) IGZO TFTs under positive bias stress (PBS) using TCAD and DFT simulation. Findings reveal that oxygen vacancies accumulating at the channel/passivation layer interface and within the channel under PBS lead to negative threshold voltage shifts and reduced mobility. Additionally, higher tail state densities contribute to a positive Vth shift. These results provide important insights into the defect-related reliability of EMMO IGZO TFTs, guiding the design of more reliable devices.

## 1. Introduction

Thin-film transistors (TFTs) are indispensable as active matrix switching devices in flat panel displays, including liquid crystal displays (LCDs) and organic light-emitting diodes (OLEDs). In recent years, significant research has been dedicated to amorphous oxide semiconductors (AOS) as potential replacements for amorphous silicon (a-Si) in TFT applications [1,2]. In 2004, Nomura et al. introduced amorphous indium gallium zinc oxide (a-IGZO) TFTs on flexible substrates, sparking widespread interest in a-IGZO technology [3]. This material is particularly attractive due to its low-temperature deposition process, high electron mobility, large-area compatibility, and excellent uniformity, making it a strong candidate for next-generation display technologies. From a manufacturing perspective, a-IGZO’s compatibility with low-temperature processes is essential for the development of flexible and large-area electronics. Furthermore, its higher electron mobility compared to a-Si offers advantages in faster switching speeds and improved device performance. However, achieving long-term stability remains a challenge, primarily due to defect states within the IGZO channel and issues related to interface passivation. These defects can degrade device performance over time, leading to variations in critical parameters, such as threshold voltage and mobility, which are vital for reliable display applications [4,5,6].

This work investigates the elevated-metal metal-oxide (EMMO) structure in TFTs, which incorporates the etch stop (ES) layer without additional masks by elevating the source–drain electrodes onto the passivation layer [7,8,9]. This integration streamlines the manufacturing process, eliminating extra masks, enhancing process efficiency, and improving channel quality [10]. Despite these advancements, EMMO IGZO TFTs encounter several reliability challenges, particularly under positive bias stress (PBS) and positive bias illumination stress (PBIS), along with potential interactions with temperature [11,12,13]. Under PBS conditions, EMMO TFTs exhibit a negative threshold voltage (Vth) shift, in contrast to the usual positive shift associated with charge trapping [14,15,16]. This study examines the device behavior under PBS through a combination of TCAD and DFT simulations, focusing on factors such as IGZO thickness, channel length, and the density of states. The analysis reveals potential mechanisms affecting device performance and reliability, providing valuable insights for the optimization of EMMO IGZO TFTs.

## 2. First-Principles Simulation

**Figure 1:**
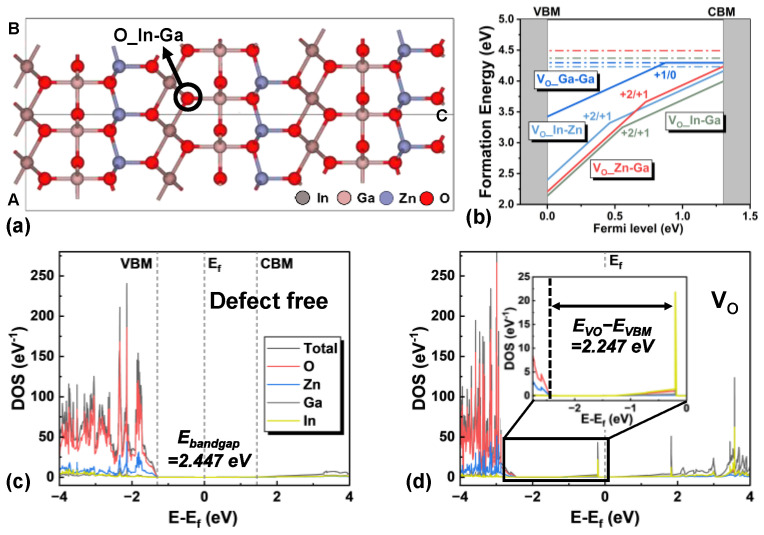
(**a**) Atomic structure of c-IGZO supercell, where the position of energetically favorable oxygen vacancy is marked. (**b**) Vacancy formation energies of four different oxygen which are bonded to indium, gallium, and zinc atoms (solid: charged states, dash: neutral states). Density of states distribution projected on the types of elements for (**c**) defect-free c-IGZO and (**d**) c-IGZO with a V_O_. The inset of (**d**) illustrates the position of V_O_ defect level.

Like other amorphous oxide semiconductors (AOS), IGZO is inherently an n-type semiconductor, and its conductivity is primarily attributed to oxygen vacancies (V_O_) [17]. V_O_ can act as an electron donor, described by the reaction V_O_ = V_O_^2+^ + 2e^−^ [18,19]. To investigate the density of states (DOS) distribution of VO, first-principles calculations were performed using density functional theory (DFT) implemented in QuantumATK [20,21]. The Perdew–Burke–Ernzerhof (PBE) exchange-correlation function within the generalized gradient approximation (GGA) and the SG15 optimized norm-conserving Vanderbilt pseudopotential (ONCVPSP) were employed for all calculations. A density mesh cutoff of 140 Har and a force tolerance of 0.01 eV/Å were set for structural optimization. As a starting point, an InGaZnO_4_ crystal (c-IGZO) 2 × 2 × 1 supercell structure containing 84 atoms was constructed, as shown in Figure 1a. The lattice type of c-IGZO is hexagonal and the space group is R3m. The Brillouin zone was sampled using a 2 × 2 × 1 k-point grid. The formation energies of four different oxygen vacancies are displayed in Figure 1b. The vacancy of oxygen bonded to gallium (Ga) atoms exhibited relatively higher formation energy compared to the other three types, suggesting that the latter vacancies are more likely to form. Consequently, vacancies of oxygen bonded to both indium and gallium (O_In-Ga) were selected for the DOS analysis. To obtain a more accurate defect level within the bandgap, the hybrid functional of Heyd, Scuseria, and Ernzerhof (HSE) was used for the exchange-correlation potential, with a finer 4 × 4 × 2 k-point sampling applied in the DOS calculation. The DOS results for defect-free c-IGZO and c-IGZO containing a V_O_, projected onto different atomic types, are illustrated in Figure 1c,d. For the defect-free structure, a bandgap (E_bandgap,c_) of 2.447 eV was obtained, which is significantly larger than the 0.72 eV bandgap calculated using the PBE method. The V_O_-induced defect level (E_VO,c_) was found to be distributed 2.247 eV above the valence band maximum (VBM), with a composition similar to that of the conduction band minimum (CBM). The position of the V_O_ defect level (E_VO,c_/E_bandgap,c_ = 0.918) aligns well with the DOS model used in the TCAD simulation, verifying the validity of this model [14].

**Figure 2:**
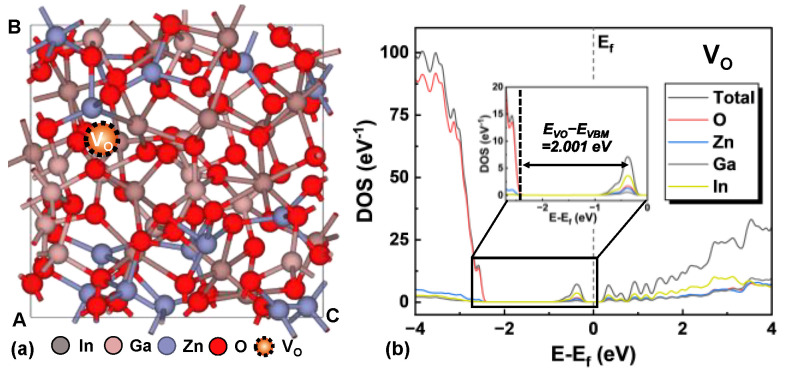
(**a**) Atomic structure of a-IGZO with a V_O_. (**b**) Density of states distribution projected on the types of elements for a-IGZO with a V_O_. The inset illustrates the position of V_O_ defect level. The Gaussian smearing is used with a width of 0.05 eV.

For further analysis of the amorphous IGZO (a-IGZO) used as the active layer in TFTs, an amorphous structure consisting of 105 atoms with the stoichiometric proportion of In:Ga:Zn:O = 1:1:1:4 was constructed using melt-and-quench ab initio molecular dynamics (MD). The DFT optimization and DOS calculation were performed using the same methods as for c-IGZO, with k-points set to 3 × 3 × 3 and 5 × 5 × 5. The bandgap of a-IGZO (E_bandgap,a_) was calculated to be 2.191 eV, closely matching previous calculations as the tail states may extend to approximately 0.4–0.6 V in the gap [22]. After constructing the defect-free a-IGZO structure, a structure containing a V_O_ defect was built, as shown in Figure 2a. Furthermore, as shown in Figure 2b, the V_O_-induced defect level of a-IGZO (E_VO,a_) was found to be located at an energy level of 2.001 eV above the VBM, with E_VO,a_/E_bandgap,a_ = 0.913. This result further confirms that the aforementioned DOS model is suitable for describing a-IGZO TFTs [14].

## 3. TCAD Simulation Methods

**Figure 3:**
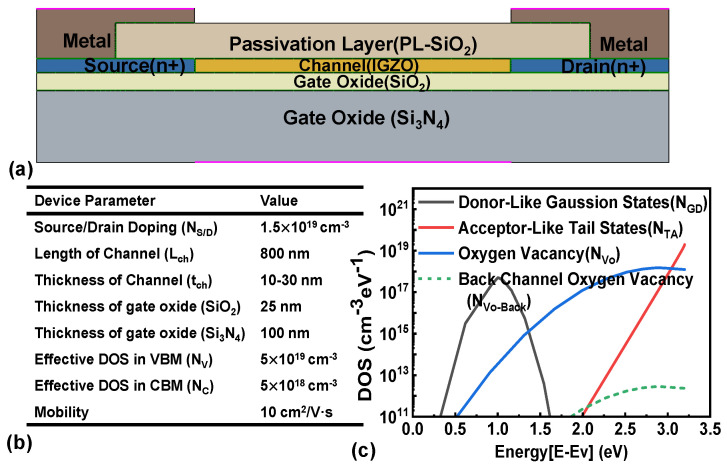
(**a**) EMMO IGZO TFTs structure and (**b**) key parameters used for simulation. (**c**) Density of states for IGZO channel.

The EMMO structure was modeled using TCAD simulations, as shown in Figure 3a. To accurately represent the device’s behavior, various physical models were implemented, including Fermi statistics, doping-dependent high-field saturation mobility, effective intrinsic density, Shockley–Reed–Hall (SRH) recombination, Auger recombination, the drift-diffusion model, trap models [23], and an interfacial mobility model. Additionally, a thin-layer mobility model and a density gradient function were incorporated to account for the characteristics of thin channel layers. For IGZO films with a channel thickness of T_ch_ = 2 nm, the lowest mobility values were found to correlate strongly with poor interfacial flatness and thickness uniformity [24]. To address this, the thin-layer mobility model was introduced. This model is specifically designed for devices with channel layers only a few nanometers thick, where geometric quantization significantly impacts mobility, rendering traditional field-dependent interface models insufficient. The thin-layer mobility model is applied alongside the Lombardi model to more accurately describe the mobility characteristics of ultra-thin layers as shown in Equations (1) and (2). Additional contributions from the normal field-dependent interface model are used in conjunction with the area. u_t1_ represented as a combination of thickness fluctuation scattering (u_tf_), surface phonon scattering (u_sp_), bulk phonon scattering (u_bp_), and surface roughness scattering (u_sr_). x is the distance from the interface. l_crit_ is a fitting parameter and refers to the critical length scale.

(1) $$\frac{1}{u_{t1}}=\frac{D}{u_{tf}}+\frac{D}{u_{sp}}+\frac{D}{u_{bp}}+\frac{D}{u_{sr}}$$

(2) $$D=e^{-\frac{x}{lcrit}}$$

It is worth noting that the metal electrode isolation induces a heavily n-type region in the source and drain areas, necessitating the inclusion of donor-like defects in these regions for comprehensive simulation. In our simulation setup, the gate oxide comprises a 100 nm Si_3_N_4_ layer and a 25 nm SiO_2_ layer, with SiO_2_ also used as the passivation layer. The equipment parameters are illustrated in Figure 3b. Defect states within the bandgap typically follow a Gaussian energy distribution centered around a specific energy level. The total density of states (DOSs) in amorphous oxide semiconductors is represented as the superposition of multiple distributions. Tail acceptor and donor states (g_TA_ and g_TD_) are characterized by their density at the valence band edge (N_TA_ and N_TD_) and their characteristic energy widths (W_TA_ and W_TD_). The E_GA_ and E_GD_ are the corresponding peak energy. Deeper states, whether acceptors or donors (g_GA_ and g_GD_), are described with a greater characteristic width and a lower density. The interface between materials, depending on the specific material and processing methods, typically presents significant and varying trap densities [25,26].

(3) $$g(E)=g_{TA}(E)+g_{TD}(E)+g_{GA}(E)+g_{GD}(E)$$

(4) $$g_{TA}\left(E\right)=N_{TA}exp\;(\frac{E-Ec}{W_{TA}})$$

(5) $$g_{TD}\left(E\right)=N_{TD}exp\;(\frac{Ev-E}{W_{TD}})$$

(6) $$g_{GA}\left(E\right)=N_{GA}exp\;[-({\frac{E_{GA}-E}{W_{GA}})}^{2}]$$

(7) $$g_{GD}\left(E\right)=N_{GD}exp\;[-({\frac{E-E_{GD}}{W_{GD}})}^{2}]$$

## 4. Simulation Results and Discussion

### 4.1. The Influence of Changes in Channel Length and Thickness

The influence of channel thickness (t_ch_) and length (L_ch_) on transistor behavior is discussed in this work. When the thickness of IGZO is less than 10 nm, we need to consider the influence of quantum effects on the transistor, so we adopted density gradient model, surface roughness scattering, and phonon scattering. The quantum potential factor Λ was inserted as a correction term into the traditional drift diffusion model to obtain the corrected drift diffusion equation, written as follows [27,28,29,30]:

(8) $$e\vec{j}=-uk_{B}T\nabla n-un\nabla (\emptyset -\Lambda )$$

(9) $$\Lambda =-\frac{\gamma h^{2}}{12m}[\nabla^{2}log\;n+\frac{1}{2}{\left(\nabla log\;\left(n\right)\right)}^{2}]$$

where n is the electron density, h is the Planck constant, γ is the fitting factor, and m is the effective mass of the electron. IGZO with different thicknesses will also introduce different levels of defect density [31,32]. Considering the subgap DOS, the Poisson equation, including quantum potential, can be alternatively solved using the following equation:

(10) $$\nabla \left(\varepsilon \nabla \psi \right)=-q(p-n+N_{D}-N_{A}+\rho_{trap})$$

(11) $$\varphi_{n}=\psi -\frac{kT}{q}\ln\;\left(\frac{n}{n_{i}}\right)-\Lambda$$

where ε is the permittivity of the semiconductor layer, φ_n_ is the quasi-Fermi potential, ψ is the electrostatic potential, k is Boltzmann’s constant, $\rho_{trap}$ is the defect densities, N_D_ represents the donor concentration, N_A_ refers to the acceptor concentration, and n_i_ is the intrinsic carrier concentration. Park et al. [33] confirmed through independent spin density measurements that the volume defect value and subthreshold swing increase with the decrease in t_ch_. According to the above explanation, as the thickness of the channel decreases, the number of acceptor-like defects in the bulk defect increases.

**Figure 4:**
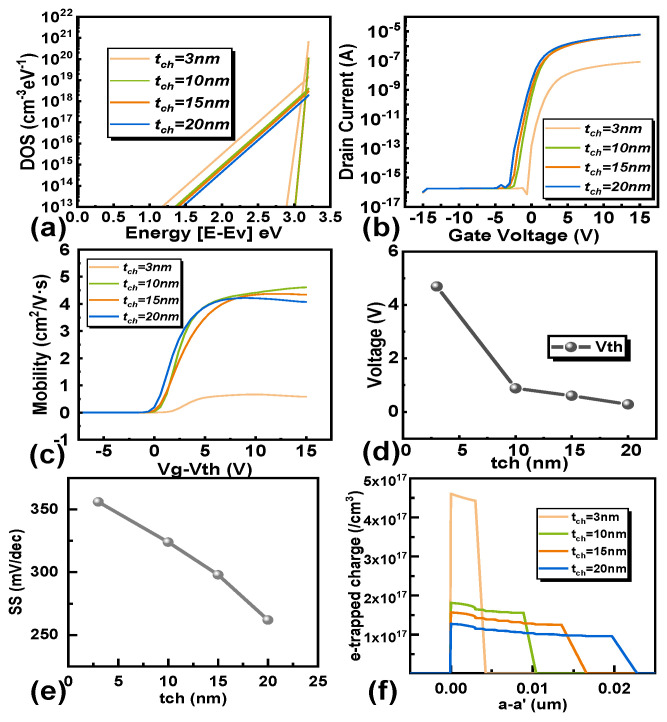
The density of states (**a**) of class acceptor defects with different channel thicknesses, (**b**) the transfer characteristic curve of the transistor, the extracted (**c**) carrier mobility, (**d**) the threshold voltage, (**e**) the subthreshold swing and (**f**) e-trapped charge which represents the number of acceptor-like defects in the channel at V_g_ = 0 V and V_ds_ = 0.2 V under different channel thicknesses.

Figure 4a shows the DOS of defects in IGZO with different channel layer thicknesses used in TCAD simulation [24]. Figure 4b shows the transfer characteristics of IGZO TFTs with different channel thicknesses. The transfer characteristics of IGZO field-effect transistors undergo a positive shift as the channel thickness decreases, and the on current also decreases accordingly. The Vth is extracted using a drain current of 100 nA, as shown in Figure 4d. Figure 4c shows the variation of field-effect mobility values with V_GS_ for devices with different channel thicknesses. The peak mobility of all IGZO field-effect transistors is related to the thickness of IGZO and the applied gate voltage. As the thickness decreases, the threshold voltage of the transistor shifts forward, indicating that in thinner transistors, a smaller surface potential is needed to deplete the entire channel layer. We can see that as the thickness of the channel decreases, the number of acceptor defects in the channel gradually increases. When the channel layer is higher than 10 nm, the effective electron mobility decreases with the increase in channel thickness. From Figure 4c, the electron mobility ranges from 4.6 cm^2^/V·s at 10 nm to 4.21 cm^2^/V·s at 20 nm. When the thickness of the channel layer is less than 10 nm, the effective electron mobility will significantly decrease. From Figure 4c, the electron mobility ranges from 4.6 cm^2^/V·s at 10 nm to 0.64 cm^2^/V·s at 3 nm. According to TCAD simulation, under constant gate voltage, the maximum electron density in a 3 nm thick channel can reach 7.28 $\times \;10^{18}$/cm^3^, while the maximum electron density in a 10 nm thick channel can reach 6.103 $\times \;10^{17}$/cm^3^. Therefore, in the case of high electron density, the probability of electron collisions increases, leading to a decrease in electron mobility. From Figure 4d, it can be seen that as the channel thickness decreases, the threshold voltage of the transistor gradually increases. In Figure 4e, we can observe a change in the subthreshold swing (SS) of the transistor, and the switching capability of the device deteriorates. The SS increasing as the channel thickness decreases, as the equation below [24]:

(12) $$SS=\frac{kT}{q}Ln(10)(1+\frac{q^{2}t_{ch}N_{bulk}+D_{it}}{C_{ox}})$$

where q is the electron charge, k is Boltzmann’s constant, T is the absolute temperature, and N_bulk_ and D_it_ are the bulk defect density of the channel and the interfacial defect density of the GI/channel region, respectively. The e-trapped charge in Figure 4f represents the number of acceptor-like defects in the channel at V_g_ = 0 V and V_ds_ = 0.2 V. It is observed that the number of acceptor-like defects is higher in thinner channels compared to thicker channels.

**Figure 5:**
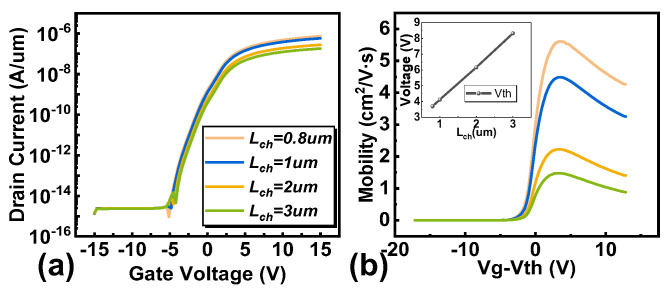
(**a**) The transfer characteristic curve and (**b**) carrier mobility under different channel lengths.

As the channel length increases, the observed field effective mobility gradually decreases (Figure 5). This reduction in mobility can be attributed to several factors, including increased scattering events due to longer channel paths, which lead to a higher likelihood of carrier interactions with impurities, phonons, and defects. Additionally, longer channels may result in higher voltage drops across the channel, further increasing scattering and reducing the effective transport of carriers. Moreover, in short-channel devices, the electric field from the gate can more effectively control the carriers, leading to higher mobility. In contrast, as the channel length increases, the control becomes less efficient, and the mobility decreases due to reduced electrostatic coupling.

### 4.2. The Impact of Defects at the Channel/PL Interface and Channel

**Figure 6:**
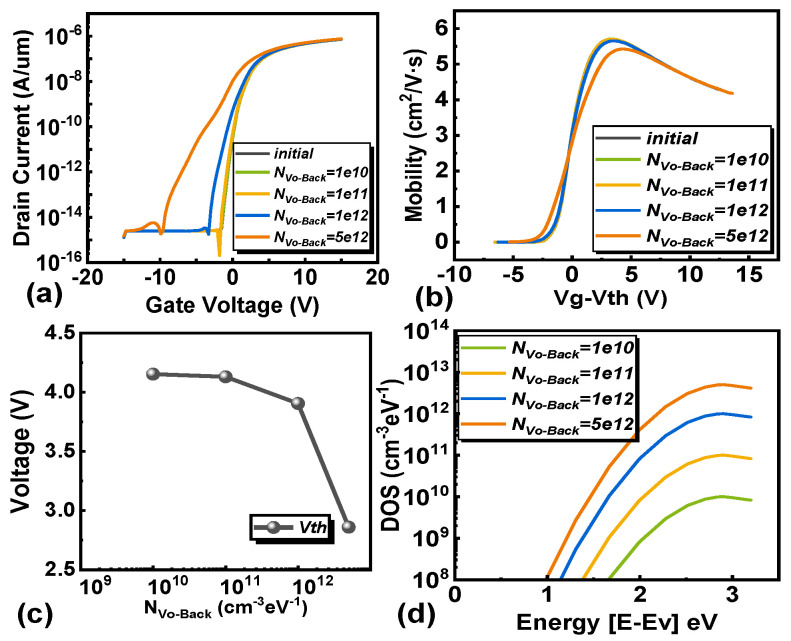
The effect of accumulated oxygen vacancy density at the channel/PL interface (N_Vo-Back_). (**a**) The transfer characteristic curves of different V_O-Back_ densities of states. The extracted (**b**) carrier mobility and (**c**) threshold voltage. (**d**) The density of states of Vo-_Back_ used in simulation.

Our research extends to evaluating the effects of oxygen vacancies at the channel/PL interface (V_O-Back_), channel oxygen vacancies (V_O_), and conduction band acceptor-like tail states (TA) on transistor behavior. Contrary to the traditional IGZO structure, the EMMO structure undergoes a negative shift under PBS conditions [34,35]. Through simulation of IGZO-TFTs with V_O-Back_, a decrease in electron mobility and a negative shift in transistor threshold voltage can be observed, as shown in Figure 6.

**Figure 7:**
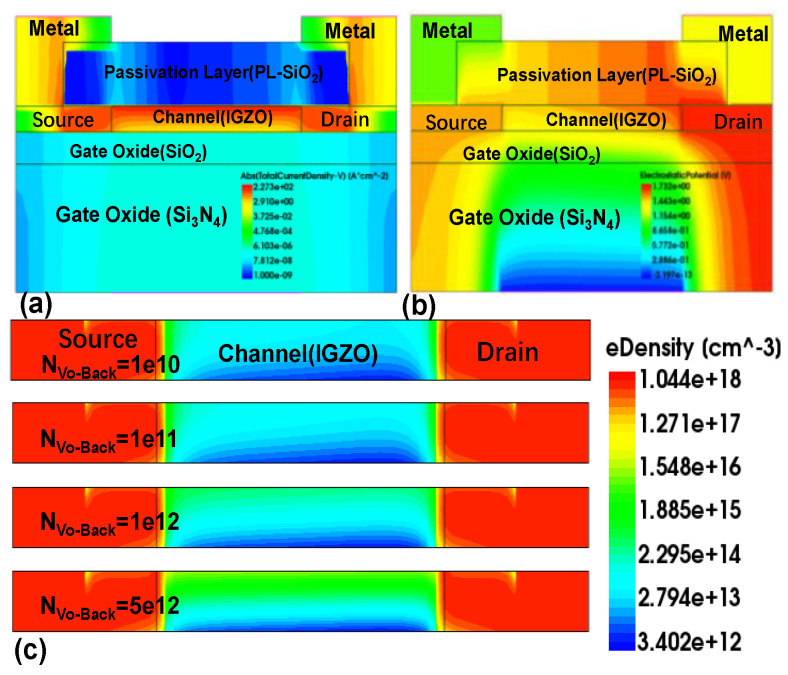
Schematic diagram of the EMMO structure after PBS simulation using TCAD: (**a**) Current density profile of the EMMO structure; (**b**) Voltage profile of the EMMO structure; (**c**) Electron density profile of the channel layer.

**Figure 8:**
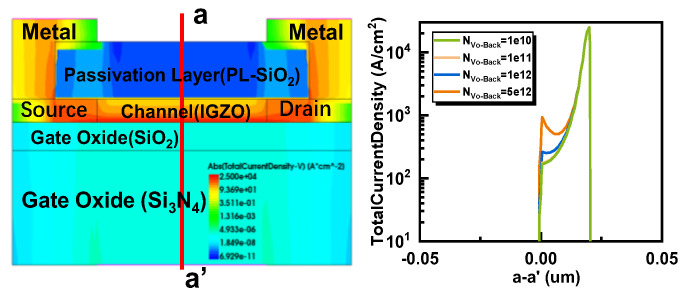
Current density distribution of different oxygen vacancy density (N_Vo-Back_) at V_g_ = 15 V. The red line a-a′ indicates the location where the current density distribution is extracted.

The root cause of this phenomenon is that under the condition of applying positive bias voltage stress, oxygen vacancies ionize into divalent oxygen vacancies (V_O_^2+^) and provide the required electrons at the back channel. The accumulation of electrons at the interface between the channel and passivation layer leads to an increase in carrier density [14,36]. It is worth noting that as the duration of the positive bias voltage stress increases, the concentration of V_O_^2+^ accumulated in the back channel increases, and the concentration of electrons contributed by it also increases. With V_g_ = 0 V, a large current density is observed on the back channel side, as shown in Figure 7a, which is attributed to the formation of V_O_^2+^. In Figure 7b, the voltage distribution within the device under PBS is illustrated. As the concentration of N_Vo-Back_ increases, the overall voltage in the channel rises, and the voltage on the side farther from the gate also increases. This results in variations in the electron concentration distribution across the channel. In Figure 7c, the distribution of electron concentrations in the channel at different donor-like defect concentrations is depicted for the back channel. The electron concentration is lower on the side closer to the gate and higher on the side farther from the gate, forming a low-resistance region. In Figure 8, the current distribution within the channel is depicted. In the on-state, a slightly higher current is observed on the side farther from the gate, indicating the presence of a low-resistance region in that area. This explains why the transistor remains in the “on” state at a gate voltage of V_g_ = 0 V. This leads to an increase in the probability of electron collisions, a decrease in mobility, and an increase in stress duration, resulting in an electron-rich channel layer different from the channel near the gate oxide layer. This may cause the back channel side to conduct prematurely, which may lead to the occurrence of the hump phenomenon. The above can be used as an explanation for the hump phenomenon generated by the EMMO structure.

**Figure 9:**
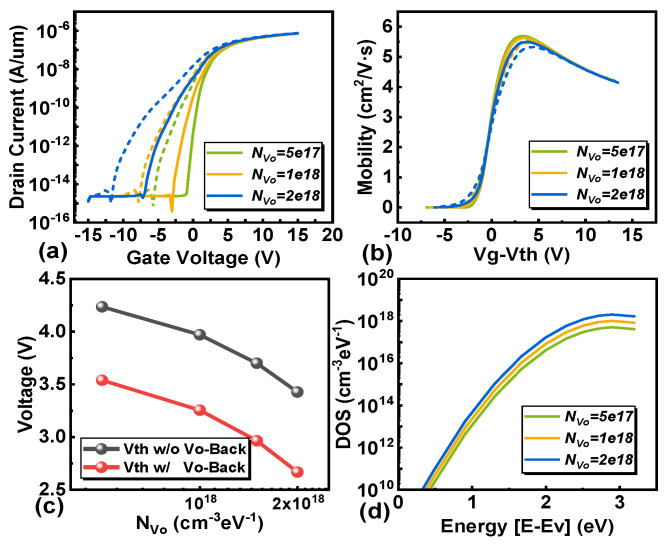
Effect of channel oxygen vacancy density ($N_{Vo}$) without (solid line) and with V_O-Back_ (dotted line). (**a**) The transfer characteristic curves of different oxygen vacancy state densities. The extracted (**b**) carrier mobility and (**c**) threshold voltage under different defect densities. (**d**) The density of states of $N_{Vo}$ in the channel.

**Figure 10:**
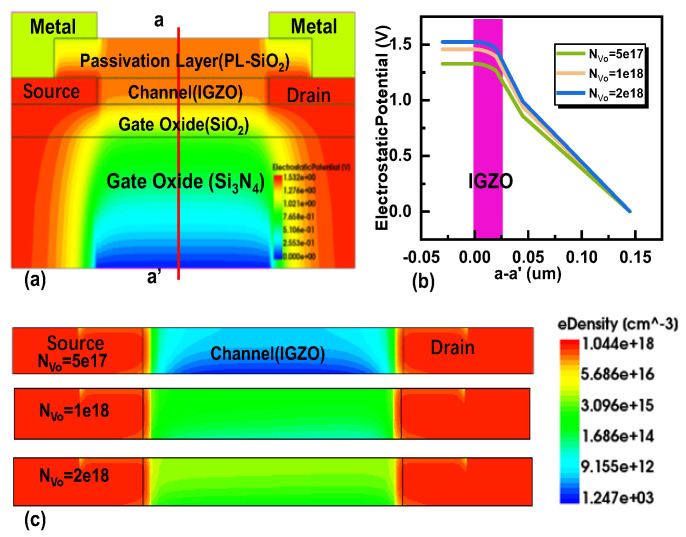
Simulation of oxygen vacancies in EMMO structural channels using TCAD at V_g_ = 0 V: (**a**) potential distribution of the EMMO structure, (**b**) voltage distribution along the a–a’ cross-section, and (**c**) electronic distribution in the channel layer.

We also investigated the effect of different concentrations of oxygen vacancies (V_O_) within the channel, as shown in Figure 9. The results indicate that as the concentration of oxygen vacancies increases under a constant gate voltage, the field-effect mobility decreases, and the threshold voltage shifts downward. Figure 10 illustrates the electron distribution of the EMMO structure at V_g_ = 0 V. As the concentration of oxygen vacancies increases, the number of ionized oxygen vacancies (V_O_^2+^) rises, leading to an increase in the total number of electrons in the channel. This, in turn, raises the potential and electric field strength within the channel layer, accelerating electron movement, increasing collision probabilities, and consequently reducing mobility. Furthermore, the electron distribution shows a higher concentration on the side farther from the gate compared to the side closer to the gate. Notably, it is observed that the density changes in channel Vo need to be significantly larger than that of V_O-Back_ to exert a discernible effect on device performance.

**Figure 11:**
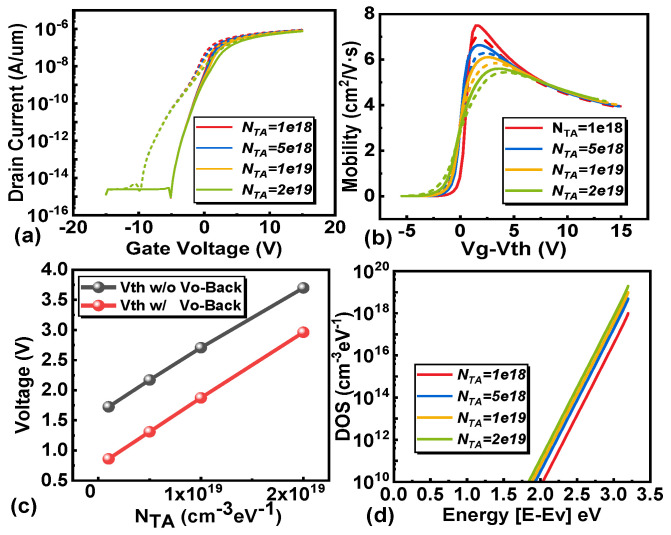
The influence of conduction band tail states density (N_TA_) without (solid line) and with V_O-Back_ (dotted line). (**a**) The transfer characteristic curves of different types of acceptor state densities. The extracted (**b**) carrier mobility and (**c**) threshold voltage under different defect densities. (**d**) The density of states of acceptor-like defects in the channel.

Furthermore, we investigate the impact of conduction band acceptor-like tail states on transistor behavior, as depicted in Figure 11. It becomes apparent that with an increase in the concentration of acceptor-like tail states, the subthreshold region of the transistor undergoes significant alteration. Notably, the subthreshold swing increases. This phenomenon arises due to the interplay within the subthreshold region, where the electron quasi-Fermi level approaches the valence band with the increasing gate voltage, leading to a rise in the number of trapped electrons and subsequently causing an increase in the subthreshold swing. Additionally, the Vth experiences a positive deviation, which may counteract the negative shift caused by channel V_O_ and V_O-Back_.

## 5. Conclusions

This study provides a comprehensive understanding of defect-related effects in EMMO IGZO TFTs under PBS conditions, highlighting the role of oxygen vacancy accumulation at the channel/passivation interface. We found that under PBS conditions, the accumulation of oxygen vacancies at the channel/PL interface and within the channel leads to a decrease in mobility and a negative shift in Vth. On the contrary, the presence of receptor-like tail states leads to a positive shift in Vth. Our findings underscore the importance of defect management for improving device reliability and suggest that targeted interface engineering could mitigate instability issues in future TFT applications.

## Data Availability

The original contributions presented in this study are included in the article. Further inquiries can be directed to the corresponding authors.
